# From Stroke to Infection: The Emerging Role of Fibrinogen-to-Albumin Ratio in Predicting Stroke-Associated Pneumonia

**DOI:** 10.7759/cureus.95203

**Published:** 2025-10-22

**Authors:** Mostafa Mubarez, Mohamed Elsayed, Yasmin Attia, Ahmed Elsaid Elsayed

**Affiliations:** 1 Neurosurgery, Faculty of Medicine, Al-Azhar University, Cairo, EGY; 2 Neurology, Al Haram Hospital, Giza, EGY; 3 Neurology, Faculty of Medicine, Benha University, Benha, EGY; 4 Neurology, Kobry El Kobba Medical Complex, Cairo, EGY

**Keywords:** acute ischemic stroke (ais), fibrinogen-to-albumin ratio (far), inflammatory biomarker, neurologic prognosis, stroke-associated pneumonia

## Abstract

Background: Stroke-associated pneumonia (SAP) is a commonly encountered complication in patients with acute ischemic stroke (AIS) and is generally manifested within the first week following stroke onset.

Aims: The objectives of this study were to determine the association of the fibrinogen-to-albumin ratio (FAR) with SAP in AIS and to examine the predictive and prognostic utility of FAR for the development of SAP.

Methods: We performed a prospective cohort study involving 44 men with AIS admitted to the stroke unit within 24 hours of onset and followed up for two weeks. Based on clinical outcomes, individual patients were classified into SAP and non-SAP groups, in addition to another 22 age-matched healthy controls. Clinical characteristics, laboratory data, and outcomes were compared for differences, and receiver operating characteristic (ROC) curves were also constructed to observe the predictive capacity of FAR.

Results: FAR uniquely distinguished the SAP group from controls with a 95.5% sensitivity and a 100% specificity at a cutoff of 0.079 (area under the curve (AUC)=0.997; p<0.001). FAR also distinguished AIS-only patients from controls with an 81.8% sensitivity and a 95.5% specificity at a cutoff of 0.072 (AUC=0.946; p<0.001).

Conclusion: FAR is a simple, robust biomarker and may independently predict the risk of SAP in AIS patients. Its very high discriminative accuracy for distinguishing SAP from both non-SAP stroke and healthy controls supports its rationale in early risk stratification and prediction. Usage of FAR in clinical assessment could facilitate the early detection of such patients, allow scope for therapeutic intervention based on individual risk, and reduce SAP-related morbidity.

## Introduction

Stroke-associated pneumonia (SAP) is one of the most common, serious complications of an acute ischemic stroke (AIS), with incidence peaking in the first week after onset. The global burden of post-stroke infection is approximately 9.1% (with the incidence of pneumonia itself at 12.4% according to large-scale studies) [1]. A systematic review and meta-analysis conducted in 2024 reported that the overall incidence of SAP was 14% and identified risk factors for SAP that were independent: male sex, age, chronic obstructive pulmonary disease (COPD), dysphagia, nasogastric tube insertion, atrial fibrillation, stroke severity, diabetes, and mechanical ventilation [2]. These all highlight the multifactorial contribution to SAP and the importance of recognizing SAP as part of post-stroke consequences.

Clinically, SAP carries a heavy burden of adverse outcomes, including increased length of stay, greater functional impairment, increased disability, and mortality across 30-day and one-year thresholds [1,2]. Furthermore, those who develop SAP are at risk not only for pneumonia as a primary diagnosis but also for other medically significant complications, including urinary tract infections, gastrointestinal bleeding, and recurrent strokes, which collectively exacerbate morbidity [2].

As awareness of clinical prediction models advances, there is increased focus on the opportunity to develop biomarkers that would help integrate systemic inflammation, coagulation, and nutritional status as part of a risk stratification protocol. Biomarkers associated with adverse outcomes with this model of integrative response include the fibrinogen-to-albumin ratio (FAR), which is a measure that combines fibrinogen, an acute-phase reactant that represents coagulation and systemic inflammation, and albumin, a negative acute-phase protein that represents nutritional reserve and systemic illness overall. Elevated FAR has been associated with poor outcomes in various clinical entities, including large-artery atherosclerotic stroke, cervical cancer, and acute coronary syndromes [3-5].

The literature supporting FAR in AIS has developed rapidly in recent years. In 2024, a cohort study demonstrated a significant and independent association between elevated fibrinogen levels and the development of SAP in AIS patients [3]. Prior research has shown that a higher FAR is indicative of worse outcomes in acute ischemic stroke, including poorer prognosis after intravenous thrombolysis [6] and mechanical thrombectomy [7]. Additionally, in studies involving broader stroke populations, higher FAR levels were associated with poorer functional outcomes, recurrent stroke, and increased mortality at 12 months post-stroke [4,5].* *When viewed confidently, FAR offers a relatively robust, accessible, and economical biomarker with clinical relevance for SAP risk stratification.

Although the link to SAP pathophysiology has improved significantly, in the way of preventative strategies, we have not enhanced. Failed trials of prophylactic antibiotic therapy have produced null changes in the incidence of SAP or how patients progress following stroke [8,9]. The measures currently undertaken, e.g., early dysphagia screening and oral care and positioning of stroke patients, have only scratched the surface of the direct issue, but they have lowered the risk without removing it. For the above reasons, identifying biomarkers that are considered reliable, such as FAR, perhaps opens the door to more accurately predict SAP risk, potentially offering a prevention pathway.

Therefore, the present study sought to investigate the association of FAR with SAP in patients with AIS and, as an additional goal, to further investigate the predictive value and prognostic capabilities overall of FAR as a risk identifier within the AIS cohort.

## Materials and methods

Study design and setting

This was an analytical comparative follow-up study that took place in the Neurology Department of Kobry El Kobba Medical Complex, Cairo, Egypt, and lasted a total of nine months. 

Patient population

Eligible patients included participants who were selected based on the inclusion and exclusion criteria. Inclusion criteria were as follows: adults >18 years of any sex, with a newly diagnosed AIS within two weeks of symptom onset. Exclusion criteria included transient ischemic attack, a previous history of central nervous system diseases with examples of brain trauma, cerebral hemorrhage, or hydrocephalus, and any history of liver disease as defined by serum transaminase levels >2 times the elevated upper limit of normal or persistent hyperbilirubinemia in the previous six months. Participants who had had pre-existing kidney disease, defined as estimated glomerular filtration rate (eGFR) <60 ml/min/1.73 m², were ineligible.

Patient assignment

All consecutive patients arriving in the Neurology Department within 24 hours after AIS were included and clinically followed for two weeks to determine whether pneumonia developed. At the conclusion of follow-up, patients were divided into three groups: group 1 included patients who had been diagnosed with SAP (SAP group); group 2, patients with stroke not associated with pneumonia (stroke-only group); and group 3, an age- and sex-matched control group with no history of stroke, pneumonia, chronic illness, or coagulopathy.

Sample size

A total of 160 patients were initially screened for eligibility. In total, 66 patients were included, including 22 patients in all three groups (AIS, SAP, and control). Sample sizes were calculated based on the discriminative power of FAR to identify SAP in Kalra et al. [8]. Originally, 66 patients were needed to provide 80% power to identify the difference of 0.21 between the area under the receiver operating characteristic (ROC) curve under the alternative hypothesis (0.71) and the null hypothesis (0.50) below the null hypothesis using a two-sided z-test at α=0.05.

Procedures

All patients with AIS were admitted to the neurology intensive care unit and had a full clinical evaluation with radiological confirmation of stroke. Patients were followed clinically for two weeks to determine pneumonia development. FAR was calculated twice, first at the time of admission and a second calculated after two weeks or when pneumonia developed, to determine the potential predictive role of FAR in SAP.

Clinical and laboratory assessments

All participants underwent complete history-taking, which was obtained by reviewing medical records and interviewing the patient. We undertook a complete clinical assessment, which included a thorough clinical examination that accounted for systemic findings (e.g., liver positive, spleen and lymph node enlargement). Radiological investigations were also done to confirm the stroke and pneumonia diagnoses. Clinical laboratory tests comprised standard investigations that included complete blood count (CBC), prothrombin time (PT), activated partial thromboplastin time (aPTT), international normalized ratio (INR), and D-dimer. Supplementary laboratory results included laboratory work for serum fibrinogen and albumin twice: once at entry to the study and then twice after two weeks or when pneumonia recurred.

Ethical considerations

The ethical application for the study was reviewed and approved by the Institutional Review Board of the Armed Forces College of Medicine (approval number: 269). The aim and purpose of the study, and all laboratory and radiological procedures, were all made clear to the participants. Prior to being included in the study, every patient was informed of the risks and completed a written informed consent process. At the conclusion of the study, participants were informed of their results, and appropriate recommendations and treatment were given.

## Results

Demographic characteristics

A total of 66 participants were enrolled and divided evenly into three groups (control: n=22; SAP: n=22; and stroke only: n=22), with a mean age of 60.59±9.51 years in the control group, 64.27±4.21 years in the SAP group, and 62.86±5.35 years in the stroke-only group. All participants were male. No significant differences were found between groups in age or sex (p>0.05) (Table 1).

**Table 1:** Demographic characteristics among the studied groups p>0.05 is statistically non-significant; p≤0.05 is statistically significant; p≤0.01 is highly statistically significant. P1: comparison between the control group and SAP group; P2: comparison between the control group and stroke-only group; P3: comparison between the SAP group and stroke-only group X^2^: chi-squared test; F: one-way ANOVA test, post hoc analysis by Tukey test SAP: stroke-associated pneumonia; SD: standard deviation

Variable	Control (N=22)	SAP (N=22)	Stroke only (N=22)	Test statistic	P-value
Sex, male, N (%)	22 (100%)	22 (100%)	22 (100%)	χ²=0.00	1.00
Age, mean±SD (years)	60.59±9.51	64.27±4.21	62.86±5.35	F=1.66	0.19
Age, range (years)	24-71	59-71	54-72	-	-
Post hoc Tukey comparisons	-	-	-	P1=0.17 (control vs. SAP); P2=0.50 (control vs. stroke only); P3=0.76 (SAP vs. stroke only)	-

Neurological features

There was evidence of heterogeneity in stroke etiology as the stroke types differed significantly between the SAP and stroke-only groups (p=0.032). Small vessel occlusion was the most frequent stroke type in the SAP group (n=13; 59.1%) vs. in the stroke-only group (n=5; 22.7%). There was no significant heterogeneity in the subtypes of stroke or the location of the lesions in the study when assessed as stroke type to group in any comparison (p>0.05). Swallowing disturbance was significantly more common in the SAP group (n=22; 100%) vs. in the stroke-only group (n=13; 59.1%) (p=0.001).

Functional outcomes showed significant variability between groups. The median modified Rankin Scale (mRS) at discharge was also significantly higher as mRS was increased in the SAP group (mRS score at discharge was 3 (IQR: 2-4)) compared to the stroke-only group (1.5 (IQR: 1-2)) (p<0.001). The stroke severity based on the National Institutes of Health Stroke Scale (NIHSS) assessment was also significantly higher in the SAP group (median of 7 (IQR: 6-9)) vs. in the stroke-only group (median of 3 (IQR: 2-4)) (p<0.001) (see Table 2).

**Table 2:** Comparison between the studied groups regarding neurological data p>0.05 is non-significant; p≤0.05 is significant; p≤0.01 is highly significant. X^2^: chi-squared test; ^Z^_MWU_: Mann-Whitney U test; T: Student's t test; ^FET^: Fisher's exact test SAP: stroke-associated pneumonia; mRS: modified Rankin Scale; NIHSS: National Institutes of Health Stroke Scale; IQR: interquartile range; SD: standard deviation

		SAP group (N=22)		Stroke-only group (N= 22)		Test value	P-value
		No.	%	No.	%		
Stroke etiology	Atherosclerosis	9	40.9%	17	77.3%	X^2^=4.607	0.032
	Small vessel occlusion	13	59.1%	5	22.7%		
Stroke subtypes	Hemorrhagic	5	22.7%	2	9.1%	X^2^=1.53	0.412^FET^
	Ischemic	17	77.3%	20	90.9%		
Location of lesion	Brainstem	6	27.3%	6	27.3%	X^2^=0.0	1.00
	Cortical	10	45.5%	10	45.5%		
	Subcortical	6	27.3%	6	27.3%		
Vomiting	No	15	68.2%	13	59.1%	X^2^=0.393	0.531
	Yes	7	31.8%	9	40.9%		
Seizure	No	12	54.5%	9	40.9%	X^2^=0.820	0.365
	Yes	10	45.5%	13	59.1%		
Swallowing disturbance	No	0	0%	9	40.9%	X^2^=11.3	0.001^FET^
	Yes	22	100%	13	59.1%		
Discharge mRS	Median (IQR)	3 (2-4)		1.5 (1-2)		^Z^_MWU_=5.02	<0.001
	Range	2-4		1-2		-	-
NIHSS score (stroke severity)	Median (IQR)	7 (6-9)		3 (2-4)		^Z^_MWU_=5.23	<0.001
	Range	4-11		2-5		-	-

Respiratory parameters

At baseline respiratory assessment after two weeks, there were large differences between the SAP and stroke-only groups. Of the patients in the SAP group, dyspnea was experienced by all patients (N=22; 100%), but the stroke-only group experienced no dyspnea (p<0.001). Swallowing disturbance persisted in all the SAP patients (N=22; 100%), while only 13 (59.1%) of the stroke patients reported swallowing disturbance (p<0.001). The odds ratio for developing swallowing disturbance in the SAP group versus the stroke-only group was approximately 32 (95% CI: wide due to small counts), indicating a strong association between SAP and swallowing dysfunction.

Oxygen saturation values were significantly lower in the SAP group (median of 90% (IQR: 89-91)) versus the stroke-only group (median of 95.5% (IQR: 95-97)) (p=0.018) (Table 3).

**Table 3:** Comparison between the studied groups regarding baseline respiratory data after two weeks p>0.05 is non-significant; p≤0.05 is significant; p≤0.01 is highly significant. X^2^: chi-squared test; KW: Kruskal-Wallis test SAP: stroke-associated pneumonia; IQR: interquartile range

Variable	SAP (N=22)	Stroke only (N=22)	Test statistic	P-value
Dyspnea, N (%)	22 (100%)	0 (0%)	χ²=44	<0.001
Swallowing disturbance, N (%)	22 (100%)	13 (59.1%)	χ²=11.31	<0.001
Oxygen saturation, median (IQR)	90 (89-91)	95.5 (95-97)	KW=50.3	0.018
Oxygen saturation, range	88-93	95-98	-	-

Lab findings

Patients in the SAP group demonstrated a strong increase in fibrinogen (median of 569.5 mg/dL (IQR: 490-790)) at two weeks compared with patients who experienced a stroke alone (median of 365.5 mg/dL (IQR: 355-380)) (p<0.001). Similarly, albumin concentrations differed between the groups (5060 mg/dL (IQR: 4590-5350) vs. 4037.5 mg/dL (IQR: 3655-4790); p=0.002). As a result of these differences, FAR was greatly increased in the SAP group (0.132 (IQR: 0.097-0.164)) compared to the stroke-only group (0.091 (IQR: 0.078-0.102)) (p=0.031) (Table 4).

**Table 4:** Comparison between the studied groups regarding fibrinogen, albumin, and FAR after two weeks p>0.05 is non-significant; p≤0.05 is significant; p≤0.01 is highly significant. KW: Kruskal-Wallis test, post hoc analysis by Tukey test SAP: stroke-associated pneumonia; FAR: fibrinogen-to-albumin ratio; IQR: interquartile range

	SAP group (N=22)	Stroke-only group (N=22)	KW	
	Median (IQR)	Median (IQR)	Test value (KW)	P-value
Fibrinogen (mg/dL)	569.5 (490-790)	365.5 (355-380)	52.71	<0.001
Albumin (mg/dL)	5060 (4590-5350)	4037.5 (3655-4790)	13.73	0.002
FAR	0.132 (0.097-0.164)	0.091 (0.078-0.102)	48.13	0.031

Clinical outcomes

Mortality in the SAP cohort was significantly higher (N=4; 18.2%) compared to the stroke-only cohort (N=0; 0%) (p=0.03). Additionally, length of stay was longer among SAP patients (median of 13 days (IQR: 11-14)) compared to stroke-only patients (median of 10 days (IQR: 9-12)) (p=0.021) (Table 5). 

**Table 5:** Outcome data among the studied groups p>0.05 is non-significant; p≤0.05 is significant; p≤0.01 is highly significant. X^2^: chi-squared test; KW: Kruskal-Wallis test SAP: stroke-associated pneumonia; IQR: interquartile range

Outcome	SAP (n=22)	Stroke only (n=22)	Test statistic	P-value
Mortality, alive, n (%)	18 (81.8%)	22 (100%)	χ²=4.4	0.03
Mortality, died, n (%)	4 (18.2%)	0 (0%)	χ²=4.4	0.03
Length of stay, median (IQR), days	13 (11-14)	10 (9-12)	KW=34.55	0.021
Length of stay, range, days	10-17	3-15	-	-

Predictive value of FAR

ROC curve analysis was conducted to determine the predictive value of FAR for identifying SAP. The optimal cutoff value for SAP from controls was 0.079, with 95.5% sensitivity and 100% specificity (area under the curve (AUC)=0.997; p<0.001). FAR also distinguished stroke-only patients from controls at a cutoff of 0.072, with 81.8% sensitivity and 95.5% specificity (AUC=0.946; p<0.001). When differentiating SAP from stroke-only patients, the cutoff was 0.105 with 59.1% sensitivity and 95.5% specificity (AUC=0.775; p<0.001) (Table 6).

**Table 6:** Validity (AUC, sensitivity, specificity) for fibrinogen-to-albumin ratio in the prediction and detection of SAP *: statistically significant at p≤0.05 SAP: stroke-associated pneumonia; AUC: area under the curve; NPV: negative predictive value; PPV: positive predictive value

Fibrinogen-to-albumin ratio	Best cutoff	Sensitivity	Specificity	PPV	NPV	AUC	P-value
SAP group vs. control group	0.079	95.5%	100%	100%	95.7%	0.997	<0.001
Stroke-only group vs. control group	0.072	81.8%	95.5%	94.8%	84%	0.946	<0.001
SAP group vs. stroke-only group	0.105	59.1%	95.5%	93%	90%	0.775	<0.001

## Discussion

Stroke incurs significant pneumonia risk via the interaction of multiple complex, pathophysiological mechanisms. Post-stroke dysphagia is an established and powerful predictor, with one meta-analysis determining an odds ratio of nearly 10 for post-stroke pneumonia in those with dysphagia (OR 9.6; 95% CI 5.75-16.04; p<0.0001) [10].

Observational cohort data support these findings, showing dysphagia predicts aspiration pneumonia independently (OR 2.61; 95% CI 2.21-3.07; p<0.001) [11]. In addition to swallowing dysfunction, post-stroke immunodeficiency, characterized by elevated levels of interleukin-6 (IL-6), interleukin-10 (IL-10), and C-reactive protein (CRP), along with reduced levels of repulsive guidance molecule A (RGM-A), is increasingly recognized as a key factor contributing to pulmonary vulnerability in post-stroke patients. This suggests that the dysregulated inflammatory response of the immune system to brain injury may significantly increase the risk of developing pneumonia [12].

Epidemiological data show a pooled post-stroke pneumonia incidence of approximately 14% (95% CI 13-15%) and identify major risk factors including age, dysphagia, severity of the stroke, COPD, and atrial fibrillation [2]. Notably, early assessment of dysphagia has demonstrated a protective effect: dysphagia screening resulted in lower odds of pneumonia (OR 0.60; 95% CI 0.42-0.84; p=0.003), supporting the value of screening dysphagia in the stroke care pathway [13]. Together, these findings emphasize the importance of implementing systematic screening for dysphagia and monitoring immunology in stroke care to help mitigate the significant burden of pneumonia and associated harms.

In our cohort, the mean age of patients in the SAP group was 64.27±4.21 years, compared to 62.86±5.35 years in the stroke-only group. All participants were male, reflecting the military setting, and the difference in mean age was not statistically significant (p>0.05). This means we can expect to see similar ages clinically. We note that this difference in ages may not be in agreement with Jitpratoom and Boonyasiri, since the mean age was also much greater (mean ≈ 65 years) in their SAP cohort than the controls in their retrospective study of acute ischemic stroke patients performed in Thailand [14].

We note that small vessel occlusion was much more prevalent in the SAP group (N=13; 59.1%) than in the stroke-only group (N=5; 22.7%) (p=0.032). Dysphagia was noted in all SAP patients and was also significantly more prevalent than had been noted in the stroke-only group (p=0.001). The functional severity of the stroke meaningfully worsened in the SAP group (p<0.001) as measured by the NIHSS and the mRS at discharge. The results of this study align with previous studies authored by Ahmad et al., Lin et al., and Li et al., which had also reported associations between stroke subtype (most importantly small vessel disease), NIHSS score, and SAP risk [2,15,16].

In contrast, some studies did not find significant differences in NIHSS scores between patients with and without SAP. In this study, the authors concluded that stroke severity was not necessarily a differentiating factor for pneumonia risk [17].

With regard to respiratory outcomes, swallowing disturbance and dyspnea were much more prevalent in the SAP group (both p<0.001), and oxygen saturation levels were significantly lower after two weeks (p=0.018). In this way, our data align with dysphagia and respiratory compromise as important predictors for pneumonia risk after stroke. Other studies similarly reported no significant association between swallowing disturbance and pneumonia occurrence, indicating that variability in study settings and definitions may influence results.

The primary outcome of this study, that fibrinogen, albumin, and FAR were significantly different in SAP patients (p<0.001), provides further evidence for the role of FAR as a biomarker of inflammatory-coagulative imbalance in patients with SAP. Furthermore, Lin et al. reported high FAR (≥0.0977) was an independent predictor of SAP (OR=2.83; p<0.001) with sufficient sensitivity and specificity, consistent with our data [15].

In our analysis of the ROC curve, FAR was found to possess a strong discriminative capability in differentiating SAP from healthy controls and stroke-only patients. Between SAP patients and healthy controls, it was found that a cutoff value of 0.079 possessed a high sensitivity of 95.5% and a specificity of 100%, with an AUC of 0.997 (p<0.001). This near-perfect diagnostic performance shows the power of FAR as a biomarker for the detection of the inflammatory and dysmetabolic state that underlies the pathogenesis of pneumonia after acute stroke.

In comparison with SAP patients versus controls, the cutoff was just a little lower at 0.072, providing a sensitivity of 81.8% and a specificity of 95.5% (AUC=0.946). Less spectacular than the SAP versus control, they are still hugely significant and show that the stroke itself, independent of pneumonia, also causes an elevation in FAR. This result is in agreement with the hypothesis that systemic inflammatory activation is an underlying feature of acute ischemic stroke but that pneumonia further magnifies the imbalance between albumin and fibrinogen to an even more pathologically distinct level [16].

Most clinically relevant is possibly the SAP vs. stroke-only patient comparison, with a higher cutoff of 0.105 identified. Sensitivity was 59.1% at this cutoff, but specificity remained at 95.5% (AUC=0.775). This indicates that while FAR is more effective at ruling in SAP due to its high specificity, its moderate sensitivity makes it unsuitable as a standalone screen. Clinicians can therefore consider FAR as a confirmatory marker, rather than an independent predictor, particularly when combined with imaging findings and clinical risk scores.

These results are consistent with those of Lin et al., who also demonstrated that FAR is an appropriate diagnostic marker in SAP for patients with acute ischemic stroke [15].

Furthermore, recent prospective cohort studies and meta-analyses have confirmed the prognostic value of FAR in different stroke subtypes and in different patterns of complications [6], highlighting its not only diagnostic but also risk stratification value. Collectively, the data show that FAR measures both systemic inflammatory burden and nutrition-inflammation balance, two important predictors of post-stroke infection and outcome.

Strengths and limitations

The present study contributes to the growing literature examining predictors of pneumonia association with stroke by examining FAR, a novel marker that combines two routinely monitored laboratory values. A notable strength is the use of clinical and biochemical data that are routinely collected, which increases the feasibility and associated costs of implementing FAR in the care of stroke patients, particularly in low-resource settings. Furthermore, the uniformity of the patient population and standardization of the diagnostic criteria for stroke and pneumonia limit the bias caused by variability in the categorization of outcomes. 

While there are strengths in this study, there are also limitations. First, being a single-center study may limit the generalizability of the findings due to potential differences in patient characteristics, management protocols, and healthcare access across regions. Second, while we accounted for the major confounders, we cannot rule out residual confounding due to variables we were unable to measure (e.g., the patients' pre-stroke nutritional status, oral care, or microaspiration risk). Third, the observational nature of the study prevents us from establishing a cause-and-effect relationship between FAR and pneumonia association in stroke patients. Finally, while the sample size met our initial power calculation, any analysis of subgroup sample size may lack power.

## Conclusions

FAR is a simple, robust biomarker and may independently predict the risk of SAP in acute ischemic stroke patients. Its very high discriminative accuracy for distinguishing SAP from both non-SAP stroke and healthy controls supports its rationale in early risk stratification and prediction. Usage of FAR in clinical assessment could facilitate the early detection of such patients, allow scope for therapeutic intervention based on individual risk, and reduce SAP-related morbidity.
